# Child Undernutrition and Convergence of Multisectoral Interventions in India: An Econometric Analysis of National Family Health Survey 2015–16

**DOI:** 10.3389/fpubh.2020.00129

**Published:** 2020-04-22

**Authors:** Sunil Rajpal, William Joe, Rockli Kim, Alok Kumar, S. V. Subramanian

**Affiliations:** ^1^Institute of Health Management Research, IIHMR University, Jaipur, India; ^2^Institute of Economic Growth, University of Delhi Enclave, New Delhi, India; ^3^Division of Health Policy and Management, College of Health Sciences, Korea University, Seoul, South Korea; ^4^Department of Public Health Sciences, Graduate School, Korea University, Seoul, South Korea; ^5^Harvard Center for Population and Development Studies, Cambridge, MA, United States; ^6^National Institution for Transforming India (NITI Aayog), Government of India, New Delhi, India; ^7^Department of Social and Behavioral Sciences, Harvard T.H. Chan School of Public Health, Boston, MA, United States

**Keywords:** child undernutrition, stunting, underweight, convergence, multisectoral interventions, India

## Abstract

In India and worldwide, there has been increased strategic focus on multisectoral convergence of nutrition-specific and nutrition-sensitive interventions to attain rapid reductions in child undernutrition. For instance, a *Convergence Action Plan* in India has been formed to synchronize and converge various nutrition-related interventions across ministries of union and state governments under a single umbrella. Given the large variation in number, nature and impact of these interventions, this paper aims to quantify the contribution of each intervention (proxied by relevant covariates) toward reducing child stunting and underweight in India. The interventions are classified under six sectors: (a) health, (b) women and child development, (c) education, (d) water, sanitation, and hygiene, (e) clean energy, and (f) growth sector. We estimate the potential reduction in child stunting and underweight in a counterfactual scenario of “convergence” where all the interventions across all the sectors are simultaneously and successfully implemented. The findings from our econometric analysis suggests that under this counterfactual scenario, a reduction of 18.37% points (95% CI: 16.77; 19.95) in stunting and 20.26% points (95% CI: 19.13; 21.39) in underweight can be potentially achieved. Across all the sectors, women and child development and clean energy were identified as the biggest contributors to the potential reductions in stunting and underweight, underscoring the importance of improving sanitation-related practices and clean cooking fuel. The overall impact of this convergent action was relatively stronger for less developed districts. These findings reiterate a clear role and scope of convergent action in achieving India's national nutritional goals. This warrants a complete outreach of all the interventions from different sectors.

## Introduction

It is widely acknowledged that child undernutrition is a multifaceted problem (1, 2). In this regard, the UNICEF (2015) conceptual framework of the determinants of child undernutrition identifies a set of immediate, underlying and basic causes of undernutrition (3). While individual diets and risk of infections are identified as the immediate causes of undernutrition, these are shaped by household food insecurity, vulnerable living environment as well as poor health care access and practices. Further, at a meso-level, these factors are influenced by a range of social, economic, and political factors and processes. The task of addressing child undernutrition, therefore, calls for multisectoral response (4) to draw upon synergies across policies and programmes of various ministries, governmental departments, and implementing partners. Multisectoral convergence is necessary to enhance complementarity between public policy initiatives and private efforts of individuals and households to prevent child undernutrition. Convergence is identified as a critical theme in organizational theory and is synonymous to concepts such as integration, collaboration, coordination, and cooperation (5–7), which are gaining recognition in national and international forums. For instance, the Sustainable Development Goals (SDGs) acknowledges the interdependent nature of all the proposed targets and places emphasis on collaborative partnerships, policy coordination, and coherence to make global progress.

In this context, India has displayed a strong commitment to integrate multisectoral convergence into its development agenda, particularly on nutrition, health and well-being. Two recent policy initiatives exemplify this effort: (a) Prime Minister's Overarching Scheme for Holistic Nourishment (National Nutrition Mission or popularly referred to as the POSHAN *Abhiyaan*); and (b) Aspirational Districts Programme (ADP). The former was launched in 2018 with targets of reducing the prevalence of child stunting, underweight, and low-birth weight by 2% points per annum, and anemia among children by 3% point per annum. The latter was designed to streamline policy efforts to address developmental needs and priorities of 115 most disadvantaged districts in India. These aspirational districts were identified from 28 states (at least one from each state) based on a composite index comprising of 49 key performance indicators across various developmental sectors, including health and nutrition (8). Importantly, both these initiatives emphasize the need for a synergistic and convergent planning to effectively transform the nutritional landscape of India.

Despite this growing emphasis on multisectoral convergence approach, the extent of synergistic effect that may arise when all the concerned sectors simultaneously work together to reduce child undernutrition remains unclear. In developing countries, the efforts to reduce child undernutrition are usually entrusted to departments concerning health care or women and child development issues, and they remain disconnected from the policy sectors responsible for household socio-economic and environmental well-being. The potential merits of multisectoral convergence, therefore, remain unknown in the absence of empirical assessments of the prospective roles of relevant sectors for reinforced action and shared accountability.

This paper aims to quantify the potential reduction in child undernutrition that can be achieved if various sectors successfully converge in India. We hypothesize that multisectoral convergence is necessary and can be more effective than different sectors working in silos. We estimate the contribution of specific interventions classified across six developmental sectors: (a) health, (b) women and child development, (c) education, (d) water, sanitation, and hygiene, (e) clean energy, and (f) growth sector. In the Indian context, each of these sectors represent different ministries and line departments. We also estimate the potential contribution of the proposed multisectoral convergence across 115 aspirational districts of India where the impact is expected to be greater than all India. To our knowledge this is the first attempt to explore the potential effects of multisectoral convergence on child undernutrition in India. The findings can contribute toward intensified convergent action and simultaneously infuse greater accountability among vital but hitherto neglected sectors in the field of child undernutrition.

## Materials and Methods

### Survey Data and Study Population

The study draws upon the data from most recent round of National Family Health Survey (NFHS), 2015–16. The sampling frame for NFHS is based on Census 2011, enabling estimations on all available indicators for 640 districts from all states and union territories (UTs). The information on health and nutritional status of children in NFHS were obtained from a two-stage stratified random sampling frame. The primary stage unit for sample were villages and census enumeration blocks for rural and urbans areas, respectively. In the second stage, households were selected for survey from each cluster/village/block on the basis of probability systematic sampling. Overall, NFHS 2015–16 provides information for 259627 singleton children. After excluding 34,627 cases with missing information on age, sex, height and weight, and excluding children aged below 12 months and above 24 months (179,712 cases), the final analytic sample was 45,288 singleton children aged 12–23 months. We restricted the final analytic sample to children aged between 12 and 23 months because diet-related data are relevant for this age group only.

### Outcome Variables of Child Undernutrition

We focus on two anthropometric indicators of child undernutrition viz. stunting and underweight. The binary variables for child stunting and underweight were constructed based on World Health Organization (WHO) growth standards (9). The NFHS provides standard information on height and weight of child. Skilled health investigators were appointed to measure weight using digital solar-powered scales along with adjustable short measuring boards. For children aged below 24 months, recumbent length was measured (10). Age- and sex-specific z scores were calculated from raw height and weight measures using WHO growth standard (9). A child's “height for age” (or “weight for age”) is a measure of their height (or weight), relative to a standard (healthy) population of the same sex and same age (in months). It is expressed as the difference between the height of the observed child and the average height of healthy children i.e., z-scores, scaled by standard deviation (SD) of child's height of healthy population. Stunting was defined as height-for-age z-scores < -2 SD and underweight as weight-for-age z-scores < -2SD.

### Key Interventions by Sectors

The primary predictors for the analysis were various indicators that correspond to various sectors represented by different ministries and line departments in India. Table 1 presents the sector-specific covariates and indicators identified for the analysis. These 23 predictors were identified to have a direct or indirect bearing on child nutritional outcomes in prior studies (11, 12). Out of all 23 binary correlates, 19 were directly associated with five nodal ministries of the government of India (Table 1). We classified household economic well-being and maternal stature as part of a growth sector that also reflects “long-term” investments and developmental strategy. Our analysis also included the following sociodemographic variables: household head's social group (categorized as; Scheduled Castes (SCs), Scheduled Tribes (STs), Other Backward Classes (OBCs), and others) and religion (categorized as; Hindu, Muslim, and others).

**Table 1:** List of selected indicators/interventions/covariates by sector with respective nodal ministries, Government of India, 2019.

**Sector**	**Intervention/Explanatory component**	**Variables (Yes = 1/No = 0)**
Health sector (Nodal Ministry: Ministry of Health and Family Welfare)	Institutional delivery	Institutional delivery
	Antenatal Care (ANC)	4+ ANC visits
	Child immunization	Full immunization
	Vitamin supplementation	Vitamin-A supplement
	Breastfeeding counseling	Breastfed within one hour
	Iron Folic Acid Supplementation (IFA)	100+ IFA doses
	Deworming dose to children	Deworming dose
	Diarrhea among children	Diarrhea
	Cough among children	Cough
	Reproductive health	Birth Order > 3
	Anemia among women	Maternal anemia (Any)
Women and child development sector (Nodal Ministry: Ministry of Women and Child Development)	Dietary intake	Full dietary diversity
	Low birth weight	Low birth weight
	Utilization of Integrated Child Development Services (ICDS)	Any ICDS benefits received by mothers
	Utilization of Integrated Child Development Services (ICDS)	Any ICDS benefits received by children
	Child marriage	Girl child marriage
	Women Body Mass Index (BMI)	Maternal low BMI
Water, sanitation, and hygiene Sector (Ministry of Jal Shakti)	Sanitation	Improved sanitation facility
	Sanitation	Safe Stool disposal
Education sector (Ministry of Human Resource Development)	Female education	Maternal matriculation
Energy sector (Ministry of Petroleum and Natural Gas)	Cooking fuel	Clean Cooking fuel
Growth sector (Ministry of Finance/Ministry of Rural Development)	Economic well-being	Wealth quintile: third or above
	Maternal stature	Maternal height >145 cm

*Interventions/covariates are classified by their respective nodal ministry of Government of India. The construction of some variables such as full dietary diversity, improved sanitary facilities, safe, stool disposal, clean cooking fuel, 4+ ANC visits, and full immunization are in accordance with the definition provided by NFHS, 2015–16 (IIPS 2017)*.

### Statistical Analysis

The prevalence of child stunting and underweight by selected correlates were reported along with 95% confidence interval (CI hereafter) via two-way cross-tables. Logistic regression was used to assess the econometric association of child stunting (and underweight) with selected primary predictors. Multivariate logistic regression were mutually adjusted for 23 primary predictors and sociodemographic covariates.

Relative risks based on post-estimations from these regression models were used to compute *population attributable risk* (PAR) for child stunting and underweight. To elaborate, PAR (expressed as percentage) shows the proportion of child stunting and underweight that can be attributed to the selected predictor(s). PAR calculations are based on comparison of “baseline scenarios” with “counterfactual or idealistic scenarios” (13). PAR estimates assume a causal relation between the predictor and outcome of interest. A number of studies have estimated PAR and PAF (population attributable fractions) to assess the impact of interventions or risk factors on child mortality in different counterfactual scenarios (14, 15). In our analysis, PAR estimates were derived based on two different mutually adjusted scenarios. In the first scenario, all 23 primary predictors and covariates were kept the same as observed in the original sample. In the counterfactual scenario, each selected predictor was set to its best condition (either 1 or 0) one at a time while keeping all other factors unchanged in the model. The difference in the PAR between the two scenarios demonstrates the extent to which child stunting and underweight can be reduced in a hypothetical ideal situation where the selected predictor is set to its best condition. For example, hypothetically, a PAR value of 3 percent for stunting to diarrhea would indicate a 3% point reduction in child stunting that could occur in the counterfactual scenario where no child suffers from diarrhea, while keeping all other factors unchanged as observed.

The following formula is used for estimating PAR based on the relative risks derived from the logistic regression analysis:

$$\begin{array}{r} PAR=\;\frac{{\left(RR-1\right)}^{*}P_{e}}{{\left(RR-1\right)}^{*}P_{e}+1} \end{array}$$

Where, RR is the relative risks (odds), P_e_ is the prevalence of the risk factor of interest.

Child undernutrition can occur due to simultaneous effects of multiple risk factors, but the PAR for multiple risk factors cannot be estimated by adding up effects of individual interventions. In such cases, the relative risks are estimated based on conditional arithmetic mean calculated based on the predicted values for given covariates and parameter estimates (13). When computing PAR for multiple interventions simultaneously, a key assumption of the model is that all the considered covariates are uncorrelated. Here, it may be noted that inadequate dietary intake and Vitamin-A deficiency are relatively more common among the poorer than richer households. Therefore, given the potential correlation between household's economic status and other covariates, we also estimated PAR values by segregating the sample into poor (bottom two wealth quintiles) and non-poor (top three wealth quintiles) as a sensitivity analysis (15). The estimations were carried out using Stata (15.0 version) and the package “*regpar”* (16). After estimating the parameters of a logistic regression model, the statistical package “*regpar”* was used to compute, and compare the predicted prevalence means between the two scenarios along with their confidence intervals (95% CI). The 95% CIs were derived by normalizing and variance-stabilizing transformations to estimate transformed parameters (17). In this special case of computing difference between two proportions/ratios (i.e., between two scenarios/PAR), Fisher's Z transformation (17, 18) was used to normalize and compute the 95% CIs.

## Results

The all India prevalence of stunting and underweight was 42.7 and 35.1% (for children 12–23 months), respectively. The prevalence, however, varied considerably across interventions from various sectors (Table 2). In the health sector, stunting prevalence was 49% among children whose mothers had not received four or more antenatal care (ANC) visits compared to those who had completed at least four ANC visits (36%). The prevalence of stunting and underweight among home born children (53.1 and 46.6%, respectively) was about 13% points higher than those born in institutional facility (40.4 and 32.5%, respectively). In the women and child development sector, the prevalence of stunting and underweight was much lower among children who received minimum dietary diversity (37.1 and 27.9%, respectively) than those who did not (43.8 and 36.6%, respectively). In the water, sanitation, and hygiene sector, about 49.4 and 43.3% of children were stunted and underweight among households with no improved sanitation facility, whereas, it was 35.0 and 25.8%, respectively among those who had improved sanitation facility. In the energy sector, households using clean cooking fuel had relatively much lower proportion of stunted children (32.4%) than those using other fuels for cooking (47.9%). In the growth sector, among all the children from poorer households (i.e., first and second wealth quintile), about 51.8% were stunted and 45.8 were underweight. Substantial burden of stunting (34.7%) and underweight (25.8%) was observed even among children from richer households.

**Table 2:** Prevalence of child (12–23 months) stunting and underweight in India by selected intervention covariates, India, NFHS 2015–16.

	**Stunting**		**Underweight**	
**Health Sector**	**Prevalence (%)**	**95% CI**	**Prevalence (%)**	**95% CI**
Institutional delivery—yes	40.4	[39.8; 40.8]	32.5	[32.1; 33.1]
Institutional delivery—no	53.1	[52.1; 54.1]	46.6	[45.6; 47.6]
4+ ANC visits—yes	36.1	[35.4; 36.7]	28.9	[28.2; 29.5]
4+ ANC visits—no	49.1	[48.4; 49.7]	41.8	[41.2; 42.4]
Full immunization—yes	40.8	[40.2; 41.3]	33.5	[32.9; 34.1]
Full immunization—no	45.9	[45.1; 46.6]	37.7	[37.1; 38.4]
Vitamin-A supplement—yes	40.9	[40.2; 41.4]	33.9	[33.4; 34.5]
Vitamin-A supplement -no	46.1	[45.3; 46.8]	37.4	[36.6; 38.1]
Breastfed within 1 hour—Yes	41.6	[40.9; 42.3]	34.1	[33.4; 34.7]
Breastfed within 1 hour—No	43.6	[42.9; 44.2]	36.1	[35.4; 36.7]
100+ IFA –yes	36.2	[35.4; 37.1]	29.1	[28.2; 29.8]
100+ IFA—no	43.2	[42.5; 43.9]	35.9	[35.2; 36.5]
Deworming dose—yes	40.8	[39.9; 41.6]	33.6	[32.8; 34.5]
Deworming dose—no	43.5	[42.9; 44.0]	35.7	[35.2; 36.2]
Diarrhea—yes	44.6	[43.4; 45.9]	40.3	[39.1; 41.5]
Diarrhea—no	42.3	[41.8; 42.8]	34.3	[33.8; 34.7]
Cough—yes	41.7	[40.5; 42.9]	35.1	[33.9; 36.3]
Cough—no	42.8	[42.3; 43.3]	35.1	[34.6; 35.6]
Birth Order >3—yes	50.9	[50.1; 51.7]	43.5	[42.7; 44.3]
Birth Order >3—no	39.3	[38.7; 39.8]	31.7	[31.2; 32.2]
Maternal anemia—yes	43.4	[42.7; 44.0]	36.5	[35.9; 37.1]
Maternal anemia—no	39.9	[39.1; 40.6]	31.2	[30.4; 31.9]
**Women and Child Development Sector**				
Full dietary diversity—yes	37.1	[36.0; 38.2]	27.9	[26.9; 28.9]
Full dietary diversity—no	43.8	[43.3; 44.5]	36.6	[36.1; 37.1]
Low birth weight—yes	49.9	[48.6; 51.1]	46.6	[45.3; 47.8]
Low birth weight—no	41.4	[40.9; 41.9]	33.1	[32.6; 33.6]
ICDS benefits—mother—yes	43.7	[43.1; 44.3]	37.2	[36.6; 37.8]
ICDS benefits—mother—no	41.2	[40.5; 41.9]	32.2	[31.5; 32.8]
ICDS benefits—child—yes	43.5	[42.9; 44.1]	36.9	[36.4; 37.5]
ICDS benefits—child—no	41.1	[40.4; 41.9]	31.8	[31.1; 32.5]
Child marriage—yes	47.8	[47.0; 48.5]	40.3	[39.5; 41.0]
Child marriage—no	39.6	[39.0; 40.1]	31.9	[31.4; 32.5]
Low BMI—yes	48.7	[47.8; 49.6]	46.9	[46.0; 47.8]
Low BMI—no	40.2	[39.6; 40.7]	30.3	[29.8; 30.8]
**Water, Sanitation, and Hygiene Sector**				
Improved sanitary facility—yes	35.0	[34.3; 35.6]	25.8	[25.2; 26.3]
Improved sanitary facility—no	49.4	[48.8; 50.0]	43.3	[42.7; 43.9]
Safe stool disposal—yes	34.3	[33.5; 35.1]	25.8	[24.9; 26.4]
Safe stool disposal—no	46.2	[45.6; 46.7]	39.1	[38.5; 39.6]
**Education Sector**				
Maternal matriculation—yes	35.1	[34.5; 5.6]	27.5	[26.9; 28.0]
Maternal matriculation—no	53.4	[52.6; 54.1]	45.8	[45.1; 46.5]
**Energy Sector**				
Clean cooking fuel—yes	32.4	[31.6; 33.2]	23.5	[22.7; 24.2]
Clean cooking fuel—no	47.9	[47.3; 48.4]	41.1	[40.5; 41.6]
**Growth Sector**				
Poor	51.8	[51.1; 52.4]	45.8	[45.1; 46.4]
Rich	34.7	[34.1; 35.3]	25.8	[25.2; 26.4]
Maternal height >145 cm—yes	39.8	[39.4; 40.3]	25.8	[25.3; 26.4]
Maternal height >145 cm—no	62.9	[61.5; 64.2]	52.3	[50.9; 53.6]

Logistic regression estimates showed statistically significant relationship between institutional delivery and the odds of stunting (OR: 0.91; 95% CI: 0.84; 0.98) and underweight (OR: 0.90; 95% CI: 0.83; 0.97) (Table 3). Children whose mothers had four or more ANC visits during pregnancy were less likely to be stunted (OR: 0.88; 95% CI: 0.83; 0.93) and underweight (OR: 0.83; 95% CI: 0.79; 0.88). Among all the correlates classified under women and child development sector, maternal BMI, dietary diversity, low birth weight, and child marriage status of mothers were found to have statistically significant associations with stunting and underweight. For example, mothers with low BMI had significantly higher odds of having a stunted (OR: 1.31; 95% CI: 1.24; 1.39) or underweight (OR: 1.88; 95% CI: 1.77; 1.99) child. A significant association was found between child undernutrition and household's sanitation practices in the water, sanitation, and hygiene sector. For instance, compared to households without improved sanitation facilities, the likelihood of children being stunted (OR: 0.86; 95% CI: 0.81; 0.92) and underweight (OR: 0.79; 95% CI: 0.74; 0.86) was significantly lower than households with improved sanitation facility. In the education sector, maternal matriculation was significantly associated with lower odds of stunting and underweight. For the energy sector, clean cooking fuel used by households was found to have significant bearing on child's nutritional status as probability of having a stunted child was lower (OR: 0.86; 95% CI: 0.81; 0.93) for those using clean fuel for cooking. All the correlates classified under growth sector were observed to have significant associations with child stunting and underweight: poorer households had higher odds of having a stunted (OR: 1.17; 95% CI: 1.09; 1.25) or underweight (OR: 1.21; 95% CI: 1.12; 1.31) child compared to wealthier households.

**Table 3:** Logistic regression estimates regarding association between child stunting (and underweight) (12–23 months) and selected covariates, India, NFHS 2015–16.

	**Stunting**		**Underweight**	
**Health Sector**	**OR**	**95% CI**	**OR**	**95% CI**
Institutional delivery—yes (ref-no)	0.91**	[0.84;0.98]	0.90***	[0.83; 0.97]
4+ ANC visits—yes (ref-no)	0.88***	[0.83; 0.93]	0.83***	[0.79; 0.88]
Full immunization—yes (ref-no)	0.97	[0.92; 1.02]	1.01	[0.95; 1.07]
Vitamin-A supplement—yes (ref-no)	0.96	[0.91; 1.02]	1.06	[1.00; 1.13]
Breastfed within 1 h—yes (ref-no)	0.96**	[0.91; 1.01]	0.92***	[0.87; 0.98]
100+ IFA—yes (ref-no)	1.01	[0.95; 1.06]	0.94	[0.89; 1.00]
Deworming dose—yes (ref-no)	1.02	[0.97; 1.08]	1.03	[0.97; 1.10]
Diarrhea—yes (ref-no)	1.02	[0.94; 1.10]	1.16	[1.07; 1.26]
Cough—yes (ref-no)	0.95	[0.88; 1.02]	0.94	[0.86; 1.01]
Birth Order >3—yes (ref-no)	1.20***	[1.13; 1.28]	1.18***	[1.11; 1.26]
Maternal anemia (any)—yes (ref-no)	1.05	[0.99; 1.10]	1.14***	[1.07; 1.20]
**Women and Child Development Sector**				
Full dietary diversity—yes (ref-no)	0.93***	[0.87; 0.99]	0.79***	[0.74; 0.86]
Low birth weight—yes (ref-no)	1.54***	[1.44; 1.66]	2.00***	[1.86; 2.16]
ICDS benefits—mother—Yes (ref-no)	1.06	[0.99; 1.13]	1.14	[1.06; 1.23]
ICDS benefits—child—Yes (ref-no)	1.01	[0.93; 1.08]	1.06	[0.98; 1.14]
Child marriage—yes (ref-no)	1.10***	[1.04; 1.16]	1.10***	[1.04; 1.17]
Low BMI—yes (ref-no)	1.31***	[1.24; 1.39]	1.88***	[1.77; 1.99]
**Water, Sanitation and Hygiene Sector**				
Improved sanitary facility—yes (ref-no)	0.86***	[0.81; 0.92]	0.72***	[0.67; 0.77]
Safe stool disposal—yes (ref-no)	0.87***	[0.82; 0.92]	0.86***	[0.80; 0.92]
**Education Sector**				
Maternal matriculation—yes (ref-no)	0.73***	[0.68; 0.77]	0.70***	[0.65; 0.74]
**Energy Sector**				
Clean cooking fuel—yes (ref-no)	0.86***	[0.81; 0.93]	0.91***	[0.84; 0.98]
**Growth Sector/Long term factors**				
Poorer (ref-richer)	1.17***	[1.09; 1.25]	1.21***	[1.12; 1.31]
Maternal height >145 cm—no (ref-yes)	2.00***	[1.84; 2.17]	1.92***	[1.76; 2.08]

Ref refers to reference category. ORs are estimates from fully adjusted model for demographic and socioeconomic covariates. Estimates are *significant at 0.10, **at 0.05 level, and ***at 0.01 level.

(Table 4) presents PAR estimates for stunting and underweight pertaining to all 23 primary predictors along with their respective sectors. If all the concerned sectors simultaneously achieved their best scenarios (counterfactual) for all 23 primary predictors via successful interventions, a possible reduction by 18.37% points (95% CI: 16.77; 19.95) in stunting and 20.26% points (95% CI: 19.13; 21.39) in underweight can be achieved (Table 4). Across all the concerned sectors, the PAR value was the highest for women and child development sector, with a PAR value of 4.94% (95% CI: 3.56; 6.33) for stunting, and 8.92% (95% CI: 7.69; 10.14) for underweight. If both components under water, sanitation, and hygiene sector were to be reversed (i.e., improved sanitary facility and safe stool disposal), stunting could reduce by 3.83% points (95% CI: 2.81; 4.86) and underweight by 5.29% points even (95% CI: 4.31; 6.27) while keeping other factors unchanged. If all the correlates under health sector achieve universal coverage, stunting can possibly reduce by 3.71% points. If all the growth sector correlates were to achieve their counterfactual scenarios, a possible reduction of 3.34% points (95% CI: 2.63; 4.06) in stunting can be achieved. The PAR value for energy sector was notable for both stunting (PAR: 2.06; 95% CI: 1.08; 3.03) and underweight (PAR: 1.23; 95% CI: 0.24; 2.21). Substantial reductions can also come from education sector both in stunting (PAR: 2.61; 95% CI: 2.11; 3.11) as well as underweight (PAR: 2.78; 95% CI: 2.29; 3.27).

**Table 4:** Population Attributable Risk (PAR) estimates for child stunting and underweight (12–23 months) associated with selected factors, India, NFHS 2015–16.

	**Stunting**		**Underweight**	
**Health Sector**	**PAR (%)**	**95% CI**	**PAR (%)**	**95% CI**
Institutional delivery	0.31	[0.06; 0.51]	0.29	[0.07; 0.51]
4+ ANC visits	1.19	[0.68; 1.71]	1.51	[1.02; 2.01]
Full immunization	0.22	[−0.06; 0.61]	−0.03	[−0.40; 0.33]
Vitamin-A supplement	0.27	[−0.10; 0.64]	−0.34	[−0.69; 0.01]
Breastfed within 1 h	0.53	[−0.09; 1.14]	0.82	[0.24; 1.14]
100+ IFA	−0.01	[−0.84; 0.67]	0.70	[−0.03; 1.43]
Deworming dose	0.03	[−1.10; 0.490	−0.36	[−1.16; 0.42]
Diarrhea	0.06	[−0.16; 0.28]	0.39	[0.18; 0.61]
Cough	0.01	[−0.03; 0.01)	−0.18	[−0.39; 0.03]
Birth order >3	1.14	[0.77; 1.51]	0.95	[0.58; 1.31]
Maternal anemia (any)	0.61	[−0.07; 1.29]	1.46	[0.82; 2.11]
**All**	**3.71**	**[1.81; 5.15]**	**3.72**	**[2.52; 6.43]**
**Women and Child Development Sector**				
Full dietary diversity	1.35	[0.17; 2.53]	3.53	[2.42; 4.64]
Low birth weight	1.46	[1.21; 1.70]	2.12	[1.88; 2.36]
ICDS benefits—mother	−0.04	[−1.10; 1.11]	−0.88	[−1.38; −0.39]
ICDS benefits—child	−0.01	[−0.51; 0.47]	−0.31	[-0.75; 0.12]
Child marriage	0.78	[0.32; 1.23]	0.71	[0.27; 115]
Maternal Low BMI	1.83	[1.44; 2.22]	3.98	[3.60; 4.36]
**All**	**4.94**	**[3.56; 6.33]**	**8.92**	**[7.69; 10.14]**
**Water, Sanitation, and Hygiene Sector**				
Improved sanitary facility	1.69	[0.97; 2.41]	3.35	[2.65; 4.05]
Safe stool disposal	2.15	[1.23; 3.08]	2.03	[1.12; 2.94]
**All**	**3.83**	**[2.81; 4.86]**	**5.29**	**[4.31; 6.27]**
**Education Sector**				
Maternal matriculation	2.61	[2.11; 3.11]	2.78	[2.29; 3.27]
**All**	**2.61**	**[2.11; 3.11]**	**2.78**	**[2.29; 3.27]**
**Energy Sector**				
Clean cooking fuel	2.06	[1.08; 3.03]	1.23	[0.24; 2.21]
**All**	**2.06**	**[1.08; 3.03]**	**1.23**	**[0.24; 2.21]**
**Growth Sector/Long term factors**				
Richer	1.48	[0.78; 2.17]	1.76	[1.07; 2.45]
Maternal height >145 cm	1.85	[1.63; 2.06]	1.61	[1.40; 1.82]
**All**	**3.34**	**[2.63; 4.06]**	**3.37**	**[2.66; 4.07]**
**Convergence of All Sectors**	**18.37**	**[16.77; 19.95]**	**20.26**	**[19.13; 21.39]**

*Estimates in bold represent total PAR value of sector*.

In terms of the PAR associated with individual interventions within each of the sectors, low maternal BMI had the greatest PAR value estimated PAR: 1.83% (95% CI:1.44; 2.22) for stunting, PAR: 3.98% (95% CI: 3.60; 4.36) for underweight within the women and child development sector. Within the water, sanitation, and hygiene sector, a possible reduction of 2.15% (95% CI: 1.23; 3.08) in stunting and 2.03% (95% CI: 1.12; 2.94) in underweight were estimated if all the households were to practice safe stool disposal.

To account for potential correlation between household's economic status and other covariates (such as dietary diversity and Vitamin A Deficiency) (Tables S3, S4) present PAR estimates for poor and non-poor separately. If all the interventions were to be put in idealized scenario simultaneously, possible reduction in stunting among the poorer households (PAR: 25.36%; 95% CI: 20.14; 30.44) would be substantially higher than those from the non-poor households (PAR: 10.68%; 8.65; 12.71) (Table S3). In case of underweight, when considering best scenarios for all interventions simultaneously, PAR values were 32.35% (95% CI: 29.18; 35.45) for poorer households and 12.08% (95% CI: 10.63; 13.52) for non-poor households (Table S4).

We also assessed PAR estimates for stunting and underweight for 115 districts identified under the ADP (Tables S1, S2). The estimates revealed a reduction of about 23.21% (95% CI: 18.98; 27.36) in child stunting and 27.74% (95% CI: 24.71; 30.72) in underweight for 115 aspirational districts if all the components (including growth sector ones) across sectors were simultaneously assumed to be in their counterfactual best scenarios (Table S2). Across sectors, the PAR value for stunting in aspirational districts was the highest for growth sector (PAR: 5.50%; 95% CI: 02.97; 8.02) (Table S2) and for underweight the women and child development sector (PAR: 11.001; 95% CI: 7.65; 14.42) had the highest attributable components (Table S2).

## Discussion

This paper offers four salient findings. First, successful implementation of interventions across different development sectors simultaneously via convergent action can substantially reduce the burden of child undernutrition levels (i.e., an estimated reduction of 18% and 20% points in stunting and underweight, respectively). Second, sectors that have received less salience in policy discourse around child undernutrition to date have the substantial potential to accelerate reductions in child stunting and underweight. For instance, across all the concerned sectors, successful scaling up of water, sanitation, and hygiene initiatives can contribute significantly toward reductions in stunting and underweight. Similarly, notable reductions are possible by improving female education and by expansion of clean cooking fuel. Third, our findings indicate that growth sector is instrumental to bring about improvements in child undernutrition via long-term factors like household health and economic well-being. Finally, the overall impact of convergent actions on reducing both stunting and underweight was relatively higher among 115 aspirational districts than for all districts combined, thus indicating greater relevance of convergence in resource-poor settings.

The findings suggest that nutrition targets under various national and international commitments (such as SDGs or the POSHAN *Abhiyaan* in India) are indeed achievable if interventions across different sectors effectively converge. However, this will require considerable synchronization in delivery of key interventions from different line departments. Ensuring cent per cent coordination across diverse sectors can be arduous, but efforts such as formulation of Convergence Action Plan (CAP) at state, district and block/village level in India provides an opportunity to harness multisectoral synergies for improving nutritional well-being. The CAP is being implemented in India under the POSHAN Abhiyaan and has garnered considerable focus in governance of nutrition programmes (5). Convergence committees (councils) at various administrative tiers have been formed comprising of senior government officials at respective levels to synchronize nutrition-specific and nutrition-sensitive interventions. The primary responsibilities of these committees are to materialize all the convergence-related activities, including development of action plan, periodic reviews of schemes and nutrition outcomes, ground level coordination across sectors, monitoring and evaluation, and identifying and filling the gaps. The CAP is developed around six broad nutrition-linked components including infrastructure, service delivery and interventions, supply chain of nutrition rations and drugs, behavioral change, and follow-up. The convergence committees are required to undertake quarterly reviews of progress and discuss the intricacies regarding multisectoral convergence based on these components.

However, the CAP may have limited reach and capacity to address constraints around provisioning of clean cooking fuel or WASH infrastructure. Moreover, the CAP cannot leverage nutrition well-being in isolation of the growth sector. Thus, the realistic benefits of convergent action may get constrained to about one-half of the estimated potential. Furthermore, a key challenge in convergent action is to ensure a coordinated response at higher levels of decision making. In fact, it is rather convenient to organize village-level activities and meetings with the help of frontline workers from different sectors (such as health, women and child development, or rural development officials) whereas development of common financial guidelines that involves two or more-line departments is cumbersome. For example, at micro level, “Village Health, Sanitation and Nutrition Day” (VHSND) has been identified as the common platform for convergent actions under POSHAN Abhiyaan (19) to provide a common service-delivery platform by three frontline workers, i.e., *Anganwadi* Worker, Auxiliary Nurse Midwife (ANM) and Accredited Social Health Activist (ASHA). While the policy intent of VHSNDs is to deliver a range (about 31) of nutrition-specific and nutrition-sensitive interventions, studies have observed a limited role of VHSNDs, restricted to only routine immunization at the grass-root level (20–22). This has been partly attributed to lack of awareness among targeted households, and partly to the supply-side inefficiencies (ie., insufficient drugs, medical instruments, and other logistics) that warrant greater coordination at higher levels of policy implementation (23–25). Similarly, investment toward behavioral interventions should ideally go hand-in-hand with infrastructure facilities and skilled human resource for quality healthcare service delivery. Investing in all these will entail financial implications for other line departments as well.

Our findings suggest a substantial role of the water, sanitation, and hygiene sector, education sector, and energy sector in effective convergence and contribution toward potential reductions in child stunting and underweight. These are in line with previous studies asserting significant association of sanitation practices with child undernutrition (26, 27). While the ongoing efforts to build toilets under Clean India Mission (*Swachh Bharat Mission*, SBM) are necessary and much appreciated, it is equally critical to ensure the utilization of these facilities by households. Addressing other supply-side bottlenecks, such as access to water in toilets, geographical access to community toilets which are far from households—especially for female adults, and girls—needs urgent programmatic focus. Our findings also reiterate the relevance of clean cooking fuel toward reduction in child stunting and underweight. Studies have observed that the use unclean fuel for cooking causes indoor pollution and hence severely affects the health and nutrition of pregnant and lactating mothers, infants and children (28, 29). While distribution of Liquified Petroleum Cylinders (LPG) to low-income households is an important nutrition-sensitive policy initiative in India (*Pradhan Mantri Ujjawala Yojana)*, the policy should be expanded with provisions for ensuring sustained use among the new beneficiaries.

Our analysis reveals notable PAR in child stunting and underweight attributed to the long-term determinants in the growth sector, such as household well-being and maternal stature. Previous studies have asserted relatively stronger associations of child stunting with household wealth and maternal stature, compared to immediate interventions like vitamin A supplementation, early breastfeeding initiation, and dietary diversity (11, 12). While immediate interventions (both nutrition-specific and -sensitive) are necessary, they are not sufficient to achieve the full potentials in improvements of nutrition health. A robust economic environment (such as improvements in real wage, income inequality) must complement convergence efforts to achieve reductions in child undernutrition (30). For long-term recovery, it is essential that nutrition policies are supported with macro-level drivers such as rural development, financial inclusion, and sustained economic growth. Previous studies on multisectoral integration in public health discourse have also asserted the crucial role of policy entrepreneurs and mid-level actors from ministries in strengthening commitment toward improving maternal and child nutritional status in India (31–33).

The following limitations should be considered when interpreting our findings. First, the cross-sectional nature of the data from NFHS restricts us from making any inference about causality between outcome and observed correlates. Second, while estimating PAR values for several exposures (or interventions) simultaneously, the model restricts the possibility of accounting for correlation between covariates and instead assume independent effects on outcome. In this regard, we computed PAR values separately for poor and non-poor households and found substantially higher need for convergence among the poorer households. In addition, it is worth noting that when several risk factors are being taken simultaneously in the model, a complete cross classification of risk factors under consideration is done to estimate reliable PAR values (34). More sophisticated models can be used to eliminate this limitation and unravel direct and indirect pathways, which further warrants temporal (or longitudinal) data. Third, due to analytical reasons, this study was limited to children aged 12–23 months. However, this age restriction does not change the distribution of children across socioeconomic groups. Fourth, due to data-specific limitations, we could not model potential contributions of food subsidies or employment schemes such as the Public Distribution System (PDS) or Mahatma Gandhi National Rural Employment Guarantee Act (MGNREGA) of India. Finally, absence of information on various supply-side bottlenecks, such as availability in terms of geographical access to interventions as well as service providers and quality, are also important data limitations to be concerned.

In conclusion, our study provides empirical evidence supporting that multisectoral convergence is critical to bring together nutrition-specific and nutrition-sensitive interventions across different sectors to accelerate reductions in child undernutrition in India. Further improvements in programmatic design is required to ensure convergent action from key line departments such as education and clean energy as well as all-encompassing growth sectors to ensure greater action in boosting nutrition well-being.

## Data Availability Statement

The datasets generated for this study will not be made publicly available. The dataset can be downloaded from www.dhsprogram.com. The codes used for statistical analysis are available upon request.

## Author Contributions

SR, WJ, and SS conceptualized and designed the study. SR led the analysis and the interpretation of the data and wrote the first draft. WJ, RK, AK, and SS contributed to the interpretation of the data and provided critical revisions. WJ and SS provided overall supervision to the study. All authors approved the final submission of the study.

## Conflict of Interest

The authors declare that the research was conducted in the absence of any commercial or financial relationships that could be construed as a potential conflict of interest.

## References

[1] Smith LC Haddad L Reducing child undernutrition: past drivers and priorities for the post-MDG era. World Develop. (2015) 68:180–204. 10.1016/j.worlddev.2014.11.014

[2] Bryce J Coitinho D Darnton-Hill I Pelletier D Pinstrup-Andersen P Maternal and Child Undernutrition Study Group. Maternal and child undernutrition: effective action at national level. Lancet. (2008). 371:510–26. 10.1016/S0140-6736(07)61694-8 18206224

[3] UNICEF UNICEF'S Approach to Scaling Up Nutrition for Mothers and their Children. United Nations Children's Fund (2015).

[4] Rasanathan K Damji N Atsbeha T Drisse MN Davis A Dora C Ensuring multisectoral action on the determinants of reproductive, maternal, newborn, child, and adolescent health in the post-2015 era. BMJ. (2015) 351:h4213 10.1136/bmj.h4213 26371220

[5] Kim SS Avula R Ved R Kohli N Singh K van den Bold M. Understanding the role of intersectoral convergence in the delivery of essential maternal and child nutrition interventions in Odisha, India: a qualitative study. BMC Public Health. (2017) 17:161. 10.1186/s12889-017-4088-z 28153041 PMC5290617

[6] Axelsson R Axelsson SB Integration and collaboration in public health-a conceptual framework. Int J Health Plann Manage. (2006) 21:75–88. 10.1002/hpm.826 16604850

[7] Garrett JL Natalicchio M editors. Working Multisectorally in Nutrition: Principles, Practices, and Case Studies. IFPRI Research Monograph. Washington DC: International Food Policy Research Institute (IFPRI) (2010). 10.2499/9780896291812

[8] NITI Aayog Aspirational Districts: Unlocking Potentials, NITI Aayog, Government of India New Delhi (2018).

[9] World Health Organization WHO Child Growth Standards: Length/Height-for-Age, Weight-for-Age, Weight-for-Length, Weight-for-Height and Body Mass Index-for-Age: Methods and Development (2006).

[10] IIPS National Family Health Survey (NFHS-4), 2015-16: Mumbai: International Institute for Population Sciences (2017).

[11] Kim R Mejía-Guevara I Corsi DJ Aguayo VM Subramanian SV Relative importance of 13 correlates of child stunting in South Asia: insights from nationally representative data from Afghanistan, Bangladesh, India, Nepal, and Pakistan. Soc Sci Med. (2017) 187:144–54. 10.1016/j.socscimed.2017.06.017 28686964

[12] Kim R Rajpal S Joe W Corsi DJ Sankar R Kumar A Assessing associational strength of 23 correlates of child anthropometric failure: an econometric analysis of the (2016). National family health survey, India. Soc Sci Med. (2019) 20:112374 10.1016/j.socscimed.2019.112374 31345611

[13] Newson RB Attributable and unattributable risks and fractions and other scenario comparisons. Stata J. (2013) 13:672–98. 10.1177/1536867X1301300402

[14] Hoffmann R Eikemo TA Kulhánová I Dahl E Deboosere P Dzúrová D The potential impact of a social redistribution of specific risk factors on socioeconomic inequalities in mortality: illustration of a method based on population attributable fractions. J Epidemiol Commun Health. (2013) 67:56–62. 10.1136/jech-2011-200886 22760220

[15] Gakidou E Oza S Fuertes CV Li AY Lee DK Sousa A. Improving child survival through environmental and nutritional interventions: the importance of targeting interventions toward the poor. JAMA. (2007) 298:1876–87. 10.1001/jama.298.16.1876 17954539

[16] Co-operation Stata 15. Stata Cooperation, College Station, TX (2017).

[17] Newson R Confidence intervals for rank statistics: Somers' D and extensions. Stata J. (2006) 6:309–34. 10.1177/1536867X0600600302

[18] Edwardes MD A confidence interval for Pr (X < Y)-Pr (X> Y) estimated from simple cluster samples. Biometrics. (1995) 1:571–8. 10.2307/2532945 7662846

[19] Paul VK Singh A Palit S POSHAN abhiyaan: making nutrition a jan andolan. Proc Indian Natl Scie Acad. (2018) 84:835–41. 10.16943/ptinsa/2018/49452

[20] Saxena V Kumar P Kumari R Nath B Pal R. Availability of village health and nutrition day services in Uttarakhand, India. J Family Med Prim Care. (2015) 4:251–6. 10.4103/2249-4863.154667 25949976 PMC4408710

[21] Parmar A Parmar N Pandya C Mazumdar VS Process evaluation of routine immunization (RI) and growth monitoring services during mamta day (village health and nutrition day) in Sinor block of Vadodara district, Gujarat, India. Natl J Commun Med. (2014) 5:378–82. Available online at: http://www.njcmindia.org/uploads/5-4_378-382.pdf

[22] Panigrahi SK Mohapatra B Mishra K. Awareness, perception and practice of stakeholders in India regarding Village Health and Nutrition Day. J Family Med Prim Care. (2015) 4:244. 10.4103/2249-4863.154663 25949975 PMC4408709

[23] Singh R Purohit B Limitations in the functioning of village health and sanitation committees in a north western state in India. Int J Med Public Health. (2012) 2:39–46. 10.4103/2230-8598.108389

[24] Gandhi SJ Dabhi M Chauhan N Kantharia S Assessment of maternal and child health services during Mamta days in urban areas of Surat City. Int J Med Sci Public Health. (2016) 5:1199–203. 10.5455/ijmsph.2016.20102015180

[25] Chambers R Von Medeazza G Sanitation and stunting in India: undernutrition's blind spot. Econ Political Wkly. (2013) 22:15-8. Available online at: https://www.epw.in/journal/2013/25/commentary/sanitation-and-stunting-india.html

[26] Rah JH Cronin AA Badgaiyan B Aguayo VM Coates S Ahmed S Household sanitation and personal hygiene practices are associated with child stunting in rural India: a cross-sectional analysis of surveys. BMJ Open. (2015) 5:e005180 10.1136/bmjopen-2014-005180 PMC4330332 25678539

[27] Cumming O Cairncross S Can water, sanitation and hygiene help eliminate stunting? Current evidence and policy implications. Mater Child Nutr. (2016) 12:91–105. 10.1111/mcn.12258 PMC5084825 27187910

[28] Hong R Banta JE Betancourt JA. Relationship between household wealth inequality and chronic childhood under-nutrition in Bangladesh. Int J Equity Health. (2006) 5:15. 10.1186/1475-9276-5-15 17147798 PMC1702347

[29] Jose S Navaneetham K Social infrastructure and women's undernutrition. Econ Poli Wkly. (2010) Mar 27:83-9. Available online at: https://www.epw.in/journal/2010/13/special-articles/social-infrastructure-and-womens-undernutrition.html

[30] Subramanian SV Kawachi I Smith GD. Income inequality and the double burden of under-and overnutrition in India. J Epidemiol Commun Health. (2007) 61:802–9 10.1136/jech.2006.053801 17699536 PMC2660005

[31] Pelletier DL Frongillo EA Gervais S Hoey L Menon P Ngo T Nutrition agenda setting, policy formulation and implementation: lessons from the Mainstreaming Nutrition Initiative. Health Policy Plan. (2012) 27:19–31. 10.1093/heapol/czr011 21292709

[32] Gillespie S Menon P Kennedy AL Scaling up impact on nutrition: what will it take? Adv Nutr. (2015) 6:440–51. 10.3945/an.115.008276 26178028 PMC4496740

[33] Gillespie S Haddad L Mannar V Menon P Nisbett N Maternal and Child Nutrition Study Group. The politics of reducing malnutrition: building commitment and accelerating progress. Lancet. (2013). 382:552–69. 10.1016/S0140-6736(13)60842-9 23746781

[34] Rockhill B Newman B Weinberg C. Use and misuse of population attributable fractions. Am J Public Health. (1998) 88:15–9. 10.2105/AJPH.88.1.15 9584027 PMC1508384

