# Supplementary material for: Child Undernutrition and Convergence of Multisectoral Interventions in India: An Econometric Analysis of National Family Health Survey 2015–16
Source: Front Public Health. 2020 Apr 22;8:129. doi: 10.3389/fpubh.2020.00129 (PMC7188776; doi:10.3389/fpubh.2020.00129)
Supplement: Supplementary file 1 [file Table_1.DOCX]

**Supplementary**

**Table S1**: Logistic regression estimates regarding association between child stunting/Underweight (12-23 months) and selected covariates, 115 Aspirational Districts, India, NFHS 2015-16

| **Health Sector** | **OR** | **95% CI** | **OR** | **95% CI** |
| --- | --- | --- | --- | --- |
| Institutional Delivery -Yes (Ref-No) | 0.97 | [0.84;1.12] | 0.92 | [0.80;1.06] |
| 4+ ANC Visits – Yes (Ref-No) | 0.83*** | [0.73;0.94] | 0.76*** | [0.67;0.86] |
| Full Immunization -Yes (Ref-No) | 1.01 | [0.90;1.14] | 1.16 | [1.02;1.32] |
| Vitamin-A Supplement -Yes (Ref-No) | 0.96 | [0.85;1.09] | 1.03 | [0.90;1.18] |
| Breastfed within 1 hour – Yes (Ref-No) | 0.96 | [0.86;1.08] | 0.96 | [0.86;1.09] |
| 100+ IFA – Yes (Ref-No) | 1.00 | [0.88;1.14] | 0.91 | [0.80;1.04] |
| Deworming Dose – Yes (Ref-No) | 1.02 | [0.89;1.15] | 1.00 | [0.87;1.14] |
| Diarrhoea – Yes (Ref-No) | 0.98 | [0.82;1.17] | 1.34 | [1.11;1.60] |
| Cough – Yes (Ref-No) | 0.90 | [0.76;1.07] | 0.86*** | [0.72;1.02] |
| Birth Order > 3 – Yes (Ref-No) | 1.16 | [1.02;1.31] | 1.19*** | [1.05;1.36] |
| Maternal Anaemia (Any) – Yes (Ref-No) | 1.06 | [0.94;1.19] | 1.07 | [0.95;1.21] |
| **Women and Child Development Sector** |  |  |  |  |
| Full Dietary Diversity – Yes (Ref-No) | 0.88** | [0.75;1.03] | 0.75*** | [0.63;0.89] |
| Low Birth Weight – Yes (Ref-No) | 1.61*** | [1.37;1.90] | 1.80*** | [1.53;2.13] |
| ICDS Benefits - Mother – Yes (Ref-No) | 1.23 | [1.05;1.43] | 1.20 | [1.02;1.41] |
| ICDS Benefits - Child – Yes (Ref-No) | 0.95 | [0.81;1.12] | 1.01 | [0.85;1.20] |
| Child Marriage – Yes (Ref-No) | 1.11*** | [0.98;1.24] | 1.00 | [0.89;1.13] |
| Low BMI – Yes (Ref-No) | 1.21*** | [1.08;1.37] | 1.87*** | [1.66;2.12] |
| **Water, Sanitation and Hygiene Sector** |  |  |  |  |
| Improved Sanitary Facility – Yes (Ref-No) | 0.79*** | [0.68;0.91] | 0.63*** | [0.54;0.73] |
| Safe Stool Disposal – Yes (Ref-No) | 1.02 | [0.87;1.19] | 0.96*** | [0.82;1.14] |
| **Education Sector** |  |  |  |  |
| Maternal Matriculation – Yes (Ref-No) | 0.72 | [0.63;0.82] | 0.71*** | [0.62;0.81] |
| **Energy Sector** |  |  |  |  |
| Clean Cooking Fuel – Yes (Ref-No) | 0.85*** | [0.71;1.03] | 0.89** | [0.73;1.09] |
| **Growth Sector** |  |  |  |  |
| Poorer (Ref-Richer) | 1.19*** | [1.01;1.41] | 1.15*** | [0.97;1.37] |
| Maternal Height > 145cm – Yes (Ref-No) | 2.17*** | [1.84;2.57] | 2.23*** | [1.89;2.64] |

**Table S2**: Population Attributable Risk (PAR) estimates for Child Stunting and Underweight (12-23 months) associated with selected Factors, 115 Aspirational Districts, India, NFHS 2015-16

|  | **Stunting** | | | **Underweight** | |
| --- | --- | --- | --- | --- | --- |
| **Health Sector** | **PAR (%)** | **95% CI** | **PAR (%)** | | **95% CI** |
| Institutional Delivery | 0.14 | [-0.56; 0.85] | 0.41 | | [-0.29; 1.11] |
| 4+ ANC Visits | 2.31 | [0.85; 3.77] | 3.23 | | [1.73; 4.68] |
| Full Immunization | 0.01 | [-0.99; 0.81] | -1.04 | | [-1.91; 0.65] |
| Vitamin-A Supplement | 0.25 | [-0.61; 1.14] | -0.21 | | [-0.10; 0.64] |
| Breastfed within 1 hour | 0.51 | [-0.91; 1.91] | 0.41 | | [-0.94; 1.70] |
| 100+ IFA | 0.01 | [-2.01; 2.10] | 1.38 | | [-0.63; 3.41] |
| Deworming Dose | -0.24 | [-2.20; 1.71] | 0.06 | | [-1.83; 1.96] |
| Diarrhoea | -0.05 | [-0.53; 0.42] | 0.73 | | [0.26; 1.21] |
| Cough | -0.32 | [-0.83; 0.19] | -0.44 | | [-0.94; 0.05] |
| Birth Order > 3 | 1.09 | [0.13; 2.05] | 1.27 | | [0.32; 2.21] |
| Maternal Anaemia (Any) | 0.89 | [-0.87; 2.66] | 0.96 | | [-0.74; 2.67] |
| All | **4.48** | **[0.79; 8.16]** | **6.76** | | **[3.27; 10.24]** |
| **Women and Child Development Sector** |  |  |  | |  |
| Full Dietary Diversity | 2.53 | [-0.58; 5.63] | 5.08 | | [2.07; 8.08] |
| Low Birth Weight | 1.55 | [1.02; 2.08] | 1.82 | | [1.30; 2.35] |
| ICDS Benefits - Mother | -1.31 | [-2.38; -0.33] | -1.09 | | [-2.01; -0.11] |
| ICDS Benefits - Child | 0.28 | [-0.56; 1.12] | -0.03 | | [[-0.83; 0.76] |
| Child Marriage | 1.06 | [-0.17; 2.30] | 0.03 | | [-1.17; 1.23] |
| Maternal Low BMI | 1.71 | [0.66; 2.74] | 5.30 | | [4.28; 6.32] |
| All | **5.78** | **[2.17; 9.37]** | **11.00** | | **[7.65; 14.32]** |
| **Water, Sanitation and Hygiene Sector** |  |  |  | |  |
| Improved Sanitary Facility | 3.52 | [1.6; 5.69] | 6.49 | | [4.37; 8.60] |
| Safe Stool Disposal | -0.32 | [9.18; 2.53] | 0.63 | | [-2.21; 3.48] |
| All | **3.21** | **[-0.07; 6.47]** | **7.11** | | **[3.93; 10.25]** |
| **Education Sector** |  |  |  | |  |
| Maternal Matriculation | 3.64 | [2.18; 5.09] | 3.69 | | [2.26; 5.12] |
| All | **3.64** | **[2.18; 5.09]** | **3.69** | | **[2.26; 5.12]** |
| **Energy Sector** |  |  |  | |  |
| Clean Cooking Fuel | 2.91 | [-0.49; 6.31] | 2.01 | | [-1.43; 5.44] |
| All | **2.91** | **[-0.49; 6.31]** | **2.01** | | **[-1.43; 5.44]** |
| **Growth Sector** |  |  |  | |  |
| Richer | 2.65 | [0.19; 5.11] | 2.02 | | [-0.43; 4.46] |
| Maternal Height > 145cm | 2.79 | [2.22; 3.37] | 2.78 | | [2.20; 3.34] |
| All | **5.50** | **[2.97; 8.02]** | **4.81** | | **[2.31; 7.31]** |
| **Convergence All Sectors** | **23.21** | **[18.98; 27.36]** | **27.74** | | **[24.71; 30.72]** |

Estimates are derived from Post estimations from Logistic Regression

**Table S3**: Population Attributable Risk (PAR) estimates for child stunting (12-23 months) associated with selected factors, India, NFHS 2015-16

|  | **Poor** | | **Non-Poor** | |
| --- | --- | --- | --- | --- |
| **Health Sector** | **PAR (%)** | **95% CI** | **PAR (%)** | **95% CI** |
| Institutional Delivery | 0.37 | [-0.12; 0.86] | 0.22 | [0.04; 0.40] |
| 4+ ANC Visits | 2.07 | [0.98; 3.14] | 0.63 | [0.14; 1.12] |
| Full Immunization | 0.30 | [-0.37; 0.98] | 0.14 | [-0.33; 0.61] |
| Vitamin-A Supplement | 0.32 | [-0.32; 0.97] | 0.20 | [-0.24; 0.65] |
| Breastfed within 1 hour | 0.23 | [-0.78; 1.23] | 0.73 | [-0.05; 1.52] |
| 100+ IFA | -1.03 | [-2.56; 0.49] | 0.27 | [-0.52; 1.07] |
| Deworming Dose | 0.67 | [-0.75; 2.09] | -0.99 | [-2.03; 0.04] |
| Diarrhoea | 0.29 | [-0.09; 0.68] | -0.10 | [-0.37; 1.06] |
| Cough | -0.26 | [-0.64; 0.01] | -0.11 | [-0.39; 0.17] |
| Birth Order > 3 | 2.04 | [1.28; 2.78] | 0.57 | [0.19; 0.94] |
| Maternal Anemia (Any) | 0.52 | [-0.66; 1.70] | 0.64 | [-0.18; 1.47] |
| All | **5.53** | **[2.86; 8.19]** | **2.21** | **[0.53; 3.89]** |
| **Women and Child Development Sector** |  |  |  |  |
| Full Dietary Diversity | 2.70 | [0.49; 4.90] | 0.68 | [-0.66; 2.38] |
| Low Birth Weight | 1.51 | [1.12; 1.90] | 1.41 | [1.09; 1.73] |
| ICDS Benefits - Mother | -0.26 | [-0.93; 0.41] | -0.57 | [-1.41; 0.26] |
| ICDS Benefits - Child | 0.09 | [-0.50; 0.70] | -0.08 | [-0.81; 0.63] |
| Child Marriage | 0.44 | [-0.44; 1.32] | 0.95 | [0.46; 1.43] |
| Maternal Low BMI | 2.57 | [1.82; 3.31] | 1.32 | [0.91; 1.73] |
| All | **7.07** | **[4.50; 9.63]** | **3.67** | **[2.07; 5.27]** |
| **Water, Sanitation and Hygiene Sector** |  |  |  |  |
| Improved Sanitary Facility | 4.28 | [2.37; 6.18] | 0.55 | [0.07; 1.13] |
| Safe Stool Disposal | 1.94 | [-0.47; 4.36] | 1.73 | [0.92; 2.54] |
| All | **6.20** | **[3.46; 8.94]** | **2.28** | **[1.44; 3.11]** |
| **Education Sector** |  |  |  |  |
| Maternal Matriculation | 3.82 | [2.67; 4.97] | 1.54 | [1.16; 1.93] |
| All | **3.82** | **[2.67; 4.97]** | **1.54** | **[1.16; 1.93]** |
| **Energy Sector** |  |  |  |  |
| Clean Cooking Fuel | 1.55 | [-3.80; 6.90] | 1.19 | [0.59; 1.79] |
| All | **1.55** | **[-3.80; 6.90]** | **1.19** | **[0.59; 1.79]** |
| **Growth Sector / Long term factors** |  |  |  |  |
| Maternal Height > 145cm | 2.45 | [2.06; 2.85] | 1.44 | [1.20; 1.68] |
| All | **1.55** | **[-3.80; 6.90]** | **1.44** | **[1.20; 1.68]** |
| **Convergence of All Sectors** | **25.36** | **[20.14; 30.44]** | **10.68** | **[8.65; 12.71]** |

**Table S4**: Population Attributable Risk (PAR) estimates for child Underweight (12-23 months) associated with selected factors, India, NFHS 2015-16

|  | **Poor** | | **Non-Poor** | |
| --- | --- | --- | --- | --- |
| **Health Sector** | **PAR (%)** | **95% CI** | **PAR (%)** | **95% CI** |
| Institutional Delivery | 0.53 | [0.04; 1.02] | 0.12 | [-0.03; 0.29] |
| 4+ ANC Visits | 2.65 | [1.58; 3.71] | 0.81 | [0.36; 1.26] |
| Full Immunization | 0.26 | [-0.40; 0.92] | -0.23 | [-0.66; 0.20] |
| Vitamin-A Supplement | -0.58 | [-1.22; 0.04] | -0.14 | [-0.55; 0.25] |
| Breastfed within 1 hour | 0.92 | [-0.06; 1.91] | 0.67 | [-0.03; 1.39] |
| 100+ IFA | 0.44 | [-1.06; 1.95] | 0.73 | [-0.07; 1.47] |
| Deworming Dose | -0.39 | [-1.79; 1.00] | -0.33 | [-1.26; 0.60] |
| Diarrhoea | 0.80 | [0.42; 1.18] | 0.13 | [-0.11; 0.38] |
| Cough | -0.09 | [-0.46; 0.28] | -0.24 | [-0.49; 0.01] |
| Birth Order > 3 | 1.42 | [0.68; 2.16] | 0.59 | [0.25; 0.94] |
| Maternal Anemia (Any) | 2.46 | [1.29; 3.62] | 0.83 | [0.09; 1.58] |
| All | **8.32** | **[5.81; 10.82]** | **2.89** | **[1.43; 4.35]** |
| **Women and Child Development Sector** |  |  |  |  |
| Full Dietary Diversity | 4.13 | [1.97; 6.28] | 3.06 | [1.85; 4.25] |
| Low Birth Weight | 2.16 | [1.77; 2.54] | 2.11 | [1.80; 2.41] |
| ICDS Benefits - Mother | -0.91 | [-1.57; 0.25] | -0.08 | [-1.51; -0.09] |
| ICDS Benefits - Child | -0.38 | [-0.97; 0.21] | -0.26 | [-0.89; 0.36] |
| Child Marriage | 0.67 | [-0.20; 1.53] | 0.72 | [0.28; 1.17] |
| Maternal Low BMI | 5.64 | [4.90; 6.38] | 2.80 | [2.41; 3.20] |
| All | **11.20** | [8.78; 13.60] | **7.20** | **[5.91; 8.50]** |
| **Water, Sanitation and Hygiene Sector** |  |  |  |  |
| Improved Sanitary Facility | 9.44 | [7.60; 11.28] | 0.82 | [0.38; 1.27] |
| Safe Stool Disposal | 0.13 | [-2.27; 2.54] | 2.06 | [1.31; 2.80] |
| All | **9.56** | **[6.94; 12.18]** | **2.83** | **[2.08; 3.58]** |
| **Education Sector** |  |  |  |  |
| Maternal Matriculation 0.82 | 4.83 | [3.69; 5.97] | 1.31 | [0.95; 1.68] |
| All | **4.83** | **[3.69; 5.97]** | **1.31** | **[0.95; 1.68]** |
| **Energy Sector** |  |  |  |  |
| Clean Cooking Fuel | 5.90 | [0.69; 11.16] | 0.59 | [0.03; 1.14] |
| All | **5.90** | **[0.69; 11.16]** | **0.59** | **[0.03; 1.14]** |
| **Growth Sector / Long term factors** |  |  |  |  |
| Maternal Height > 145cm | 2.11 | [1.72; 2.51] | 1.28 | [1.05; 1.51] |
| All | 2.11 | [1.72; 2.51] | 1.28 | [1.05; 1.51] |
| **Convergence of All Sectors** | **32.35** | **[29.18; 35.45]** | **12.08** | **[10.63; 13.52]** |
